# Children with Autism show Atypical Preference for Non-social Stimuli

**DOI:** 10.1038/s41598-019-46705-8

**Published:** 2019-07-17

**Authors:** Catherine M. Gale, Svein Eikeseth, Lars Klintwall

**Affiliations:** 1 Department of Behavioral Science, Oslo Metropolitan University, Oslo, Norway; 2 Department of Psychology, Stockholm University, Stockholm, Sweden

**Keywords:** Paediatric research, Human behaviour

## Abstract

The present investigation describes three studies testing the hypothesis that children with Autism Spectrum Disorder (ASD) show an atypical preference for non-social stimuli. Preference for non-social and social stimuli was assessed using applications on a portable tablet computer. Twenty-eight children with ASD were matched on developmental age with the chronological age of 41 typically developing (TD) children. The non-social stimuli consisted of six different films of abstract moving geometric patterns. Social stimuli were six different films of the face of young adults (Study 1 and 3) or six films of different dogs’ faces (Study 2). When given a choice between the non-social and social stimuli, children with ASD preferred the non-social stimuli. When the human faces were replaced with dogs’ faces the participants with ASD continued to prefer the non-social stimuli. A high reinforcement value of non-social stimuli was also demonstrated when the non-social stimuli were presented alone, suggesting the preference for the non-social stimuli was not simply an avoidance of social stimuli. Whenever an infant prefers non-social stimuli over social stimuli, non-typical development in social communication and social interests may result, together with the development of high levels and frequently occurring stereotyped and repetitive behavior. These behaviors define Autism.

## Introduction

Children with Autism show Atypical Preference for Non-social Stimuli. The deficits of social communication and social interest which in part defines Autism Spectrum Disorder (ASD) have often been considered primary when attempting to understand the disorder. One example is the social motivation hypothesis, which posits that a lack of social motivation and a deficit in processing of social rewards underlie ASD^1–3^. Social motivation is characterised by “social orienting” (showing preference for the social world), “social reward” (seeking and taking pleasure in social interactions), and “social maintaining” (working to foster and maintain social bonds)^1^. Children without social motivation lack the incentives to acquire social skills such as joint attention, theory of mind, pretend play or pragmatic language^1^.

A recent systematic review of the social motivation hypothesis, however, found mixed evidence for it, with only 15 of 27 studies reviewed supporting the hypothesis^4^. Bottini suggested that a general reward sensitivity deficit hypothesis that includes non-social reward processing should replace the social motivation hypothesis. This view is heavily supported by research showing that toddlers with ASD as young as 14 months of age spend significantly more time looking at non-social geometric images as compared to children with developmental delay and typically developing children of the same age^5–7^. In addition, several studies demonstrating decreased interest in social stimuli in children with ASD also show that these children had an increased interest in non-social stimuli^8–11^.

Studies examining children’s interest in social and non-social stimuli have utilized a variety of stimuli. Social stimuli have included pictures of faces^8,12,13^, videos of faces^14^, pictures of social interaction^15^, and videos of children dancing or doing yoga^5,6^. In studies utilizing non-social stimuli, the stimuli have included pictures depicting restricted or circumscribed interests^11,16,17^, household objects^14^, toys^13^, letters^18^, inverted and reversed animations^19^ and moving geometric images^5,6^.

The present investigation describes three studies assessing children’s interest in non-social geometric moving films, testing the hypothesis that children with ASD show a preference for non-social images as identified by Pierce and colleagues^5,6^ and extending the research by (a) examining the reinforcement strength of the non-social stimuli when presented with social stimuli and alone (b) using an easily administered tablet-based application rather than advanced eye tracking technology, and (c) using other non-social and social stimuli (including non-human social stimuli).

**Figure 1:**
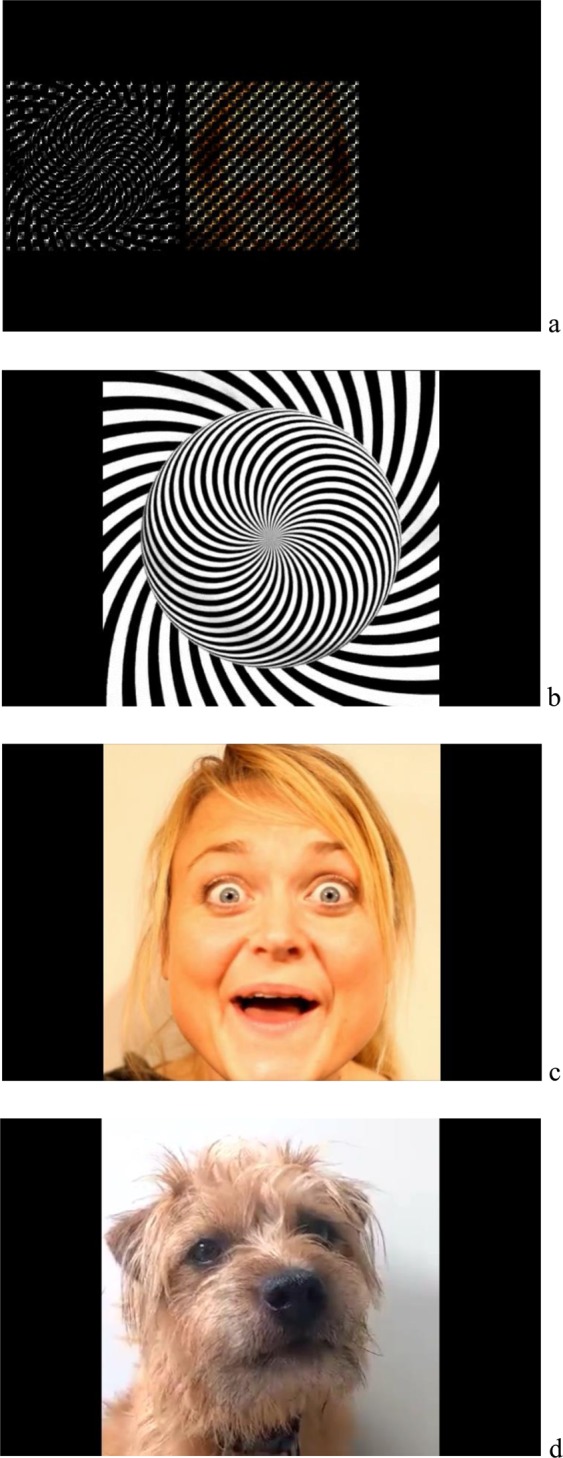
(**a**) The tablet application as it was seen by the participants. Whenever one of the two blurred stimuli was touched, it increased in size and became clearly visible for 2 seconds. (**b**) One of the six non-social geometric stimuli as it was seen after it had been touched. (**c**) One of the six social human stimuli as it was seen after it had been touched. (**d**) One of the six social nonhuman stimuli as it was seen after it had been touched.

In Study 1, non-social stimuli in the form of geometric screen-saver moving films and social stimuli in the form of films of human faces were presented in a choice arrangement on a tablet computer to two groups of participants, children with ASD and typically developing (TD) children, to identify preference (Fig. 1a–c). We predicted that the children with ASD would have a higher preference for the non-social stimuli than the TD children.

It has been suggested that children with ASD find social interaction with animals, particularly dogs, less challenging than interaction with humans and that children with ASD demonstrate a preference for dogs over humans and inanimate objects^20,21^. Therefore, preference for any non-social stimuli may be reduced when presented together with dogs’ faces (Fig. 1d). This led to the hypothesis, tested in Study 2, that children with ASD may prefer dogs’ faces to the non-social geometric stimuli thus questioning the reinforcement valence of the non-social stimuli in the absence of human faces.

Study 3 was designed to identify whether the non-social geometric stimuli maintained reinforcement value when presented alone, and to examine the reinforcement strength of the non-social and social stimuli. Reinforcement strength was assessed by how much effort the children were willing to apply to access these stimuli, using a progressive ratio reinforcement schedule. This involves an arrangement whereby the responding required to access reinforcement increases progressively during a single session^22^ or across sessions^23^. Progressive ratio schedules have been used previously to assess reinforcement efficacy for children with ASD^24,25^. Reinforcer strength is identified as the break point for the reinforcement schedule, that is, the point where the number of presses required for access to the stimuli becomes so high the child stops responding^26,27^. Study 3 was designed to assess the reinforcement strength of the non-social stimuli and social stimuli in a single stimulus arrangement in the absence of the alternative stimuli.

## Results

**Figure 2:**
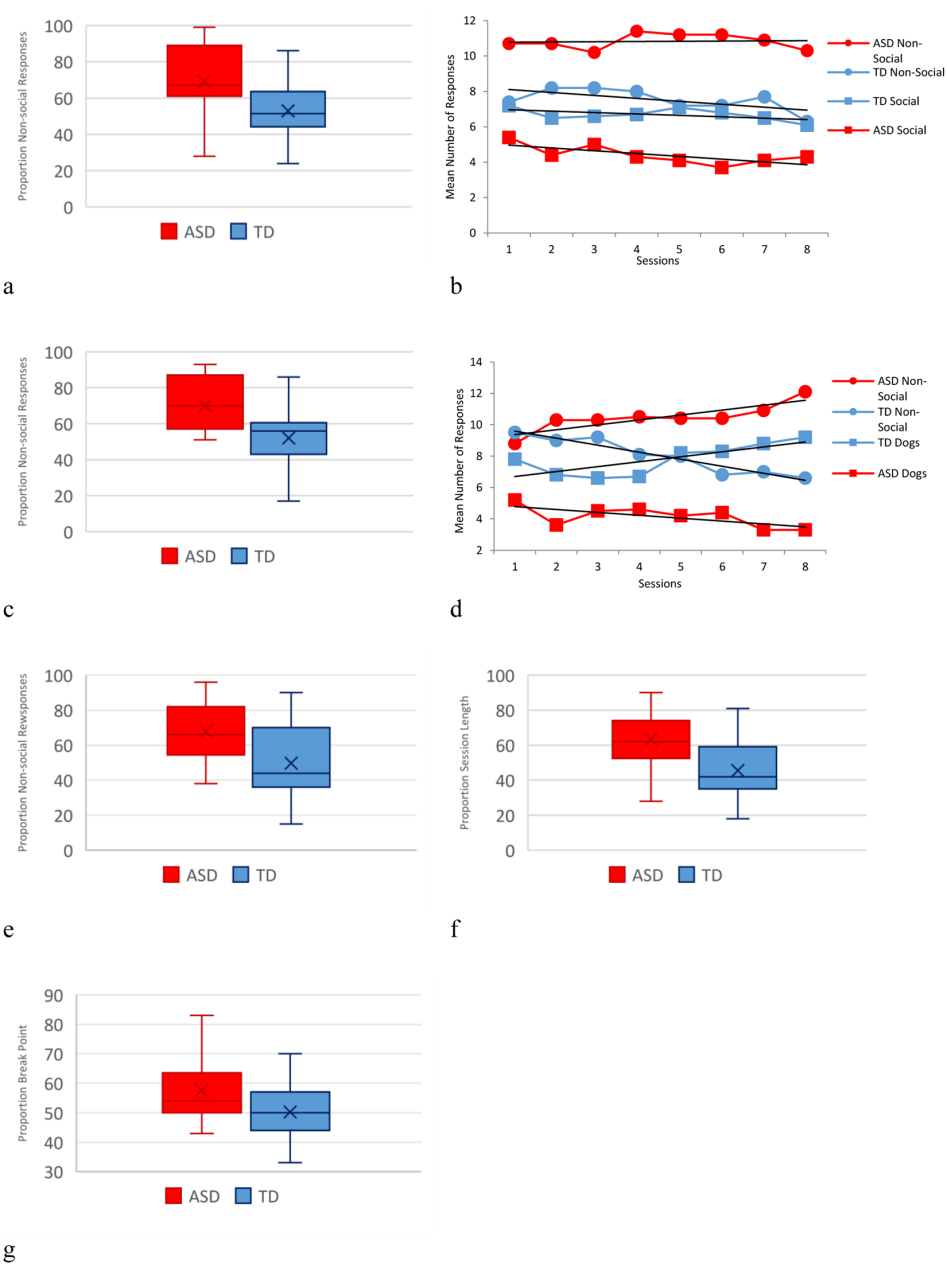
(**a**) Box and whisker plot showing proportion of responding to non-social stimuli (geometric images) when presented with social human stimuli (adults’ faces) for ASD and TD children. (**b**) Mean number of responses per session for non-social (geometric images) and human social stimuli (adults’ faces). (**c**) Box and whisker plot showing proportion of responding to non-social stimuli (geometric images) when presented with social nonhuman stimuli (dogs’ faces) for ASD and TD children. (**d**) Mean number of responses per session for non-social (geometric images) and nonhuman social stimuli (dogs’ faces). (**e**) Box and whisker plot showing proportion of responding to non-social stimuli when social and nonsocial stimuli were presented in a single choice arrangement using a progressive ratio reinforcement schedule. (**f**) Proportion of responding to non-social (geometric images) when reinforcement strength during the progressive ratio schedule was measured as session length. (**g**) Preference for non-social stimuli when reinforcement strength during the progressive ratio schedule was measured as the schedule breakpoint.

Results of Study 1 showed the mean proportion of responding to the non-social reinforcers for the participants with ASD was 69.1% (*SD* = 21.4) as compared to 52.9% (*SD* = 15.4) for the TD participants (See Fig. 2a). This difference was statistically significant (Welsch t-test, *t*(61) = 3.393; *df* = 44, *p* < 0.001), and yielded a large effect size (Cohen’s *d* = 0.871). For the children with ASD, responding was stable across sessions for the non-social reinforcers, but decreased across sessions for the social reinforcers (Fig. 2b). Hence, for the children with ASD, there was evidence of satiation for the social reinforcers, but not for the non-social reinforcers, suggesting the social reinforcers lost effectiveness across sessions whereas the non-social reinforcers did not. The TD children showed decreased responding across sessions for both the social and non-social reinforcers, suggesting both the social and non-social reinforcers reduced their effectiveness across sessions (Fig. 2b).

As the groups differed in gender proportions, the analysis was also run using only the male participants from both groups (ASD n = 21, TD n = 22). Results were similar: mean proportion non-social responding for the ASD group: 71.3% (SD = 18.2), and the TD group: 52.5% (SD = 14.2). This difference was statistically significant (Welsch t-test, *t* = 3.751; *df* = 38; *p* < 0.001), and yielded a large effect size (Cohen’s d = 1.147).

When comparing dogs’ faces with non-social geometric films (Study 2) results showed the mean proportion of responding to the non-social reinforcers for the participants with ASD was 69.9% (*SD* = 14.8) as compared to 52% (*SD* = 18.2) for the TD participants (Fig. 2c). This difference was statistically significant (*t* = 3.445, *df* = 38 *p* = 0.001) and yielded a large effect size (Cohen’s *d* = 1.085). As in Study 1, children with ASD showed evidence of stimulus satiation for the social reinforcers while TD children showed evidence of stimulus satiation for the non-social reinforcers (Fig. 2d). In contrast to Study 1, children with ASD showed increased interest in the non-social reinforcers across sessions while the TD children showed increased interest in the social reinforcers (Fig. 2d).

Because gender proportions were not similar in the two groups, analysis was rerun with males only (ASD *n* = 15; TD *n* = 11). Mean proportion non-social responding for the ASD group was: 71.1% (*SD* = 14.2), and the TD group: 44.7% (*SD* = 18.0). This difference was found to be significant (Welsch *t*-test: *t* = 4.107; *df* = 18; *p* = 0.001), and yielded a large effect size (Cohen’s *d* = 1.623).

When stimuli were presented in a single choice arrangement, on a progressive ratio reinforcement schedule (Study 3), the children with ASD preferred the non-social over the social reinforcers, as compared to the TD group: mean proportion touches to obtain the non-social reinforcers for the ASD group was 67.9% (*SD* = 16.2), and for the TD group 49.7% (*SD* = 21.2) (Fig. 2e). This difference was statistically significant (Welsch t-test, *t* = 3.075; *df* = 38; *p* = 0.004) and yielded a large effect size (Cohen’s *d* = 1.107). Running the same analysis with males only (ASD: *n* = 12, TD: *n* = 10), the results were similar: (Welsch *t*-test *t* = 2.520; *df* = 17; *p* = 0.022), and yielded a large effect size (Cohen’s *d* = 1.106).

When reinforcement strength was measured as session length (Fig. 2f), children in the ASD group preferred the non-social over the social stimuli, as compared to the TD group: mean proportion non-social duration for the ASD group was: 63.4% (*SD* = 15.6), and the TD group: 45.6% (*SD* = 16.5) (Fig. 2f). This difference was statistically significant (*t* = 3.477; *df* = 36; *p* = 0.001), and yielded a large effect size (Cohen’s *d* = 0. 0.964). Running the same analysis with males only (ASD: *n* = 12, TD: *n* = 10), the results were similar: (Welsch t-test *t* = 2.129; df = 19; *p* = 0.047), and yielded a large effect size (Cohen’s *d* = 0.916).

Finally, when reinforcement strength was measured as breakpoints (Fig. 2g), children in the ASD group preferred the non-social over the social stimuli, as compared to the TD group: mean proportion non-social breakpoints for the ASD group was: 57.4% (*SD* = 11.1), and the TD group: 50.35% (*SD* = 9.3). This difference was statistically significant (*t* = 2.196; *df* = 31; *p* = 0.036) and yielded a medium effect size (Cohen’s *d* = 0.711). Running the same analysis with males only (ASD: *n* = 12, TD: *n* = 10), the results were similar: (Welsch t-test *t* = 2.641; *df* = 20; *p* = 0.016), and yielded a large effect size (Cohen’s *d* = 1.095).

No relationships were found between any participant characteristics (developmental age, cognitive ability, autism symptoms, and adaptive behavior), and proportion of non-social responding. However, statistical power was low due to the small sample size (Table 1, supplementary materials).

**Table 1:** Participant Characteristics Study 1–3.

Study 1	ASD (*N* = 27)	TD (*N* = 40)
Gender M/F	21/6	22/18
Chronological Age (months) M (SD; Range	59.3 (18.3; 28–96)	34.5 (12.3; 14–63)
Developmental Age (months) M (SD; Range)	32.0 (10.5; 12–50)	
Cognitive Score M (SD; Range)	62.7 (24.5; 29–106)	
**Vineland Adaptive Behavior Scales**		
Adaptive Behavior Composite M (SD; Range)	65.6 (9.7; 51–84)	
Communication M (SD; Range)	70.4 (15.9; 42–104)	
Daily Living Skills M (SD; Range)	66.8 (13.2; 48–95)	
Socialization M (SD; Range)	65.3 (10.1; 51–86)	
Motor Skills M (SD; Range)	73.9 (12.0); 54–97)	
Childhood Autism Rating Scale (raw score)	36.6 (7.1; 19.5–49)	
**Study 2**	**ASD (*N*** = **19)**	**TD (*N*** = **21)**
Gender M/F	15/4	11/10
Chronological Age (months) M (SD; Range)	58.2 (21.1; 26–96)	33.6 (12.9; 17–63)
Developmental Age (months) M (SD; Range)	29.9 (10.4; 12–50)	
Cognitive Score M (SD; Range)	58.2 (21.6; 29–101)	
**Vineland Adaptive Behavior Scales**		
Adaptive Behavior Composite M (SD; Range)	65.7 (9.8; 51–85)	
Communication M (SD; Range)	68.4 (13.5; 50–97)	
Daily Living Skills M (SD; Range)	67.6 (13.3; 48–95)	
Socialization M (SD; Range)	65.7 (10.7; 53–90)	
Motor Skills M (SD; Range)	72.3 (10.6; 54–97)	
Childhood Autism Rating Scale (raw score)	37.1 (7.1; 19.5–49)	
**Study 3**	**ASD (*N*** = **17)**	**TD (*N*** = **23)**
Gender M/F	12/5	10/13
Chronological Age (months) M (SD; Range)	69.6 (17.0; 44–92)	34.9 (10.3; 17–60)
Developmental Age (months) M (SD; Range)	32.2 (10.8; 12–50)	
Cognitive Score M (SD; Range)	56.8 (23.7; 29–101)	
**Vineland Adaptive Behavior Scales**		
Adaptive Behavior Composite M (SD; Range)	62.5 (9.3; 51–83)	
Communication M (SD; Range)	67.4 (14.8; 50–104)	
Daily Living Skills M (SD; Range)	64.9 (14.2; 48–95)	
Socialization M (SD; Range)	62.9 (10.3; 51–86)	
Motor Skills M (SD; Range)	69.8 (11.5; 54–91)	
Childhood Autism Rating Scale (raw score)	38.0 (7.9; 19.5–49)	

## Discussion

Using applications designed for the purpose of this study, administered on a tablet computer, we found atypical preference for non-social stimuli in children with ASD matched on developmental age with the chronological age of TD children. When given a choice between non-social and social stimuli, children with ASD showed a stronger preference for the non-social stimuli than the TD children. When the human faces were replaced with dogs’ faces, the children with ASD continued to prefer the non-social stimuli. A strong interest in non-social stimuli was also demonstrated when the non-social stimuli were presented alone, suggesting the preference for the non-social stimuli was not simply an avoidance of social stimuli. These results demonstrate the non-social stimuli functioned as a powerful reinforcer for the behavior of the children with ASD. Moreover, across repeated sessions, the reinforcement strength of the non-social stimuli remained high for the children with ASD while decreasing for the TD children.

These results extend previous research^5,6^ by (a) examining the reinforcement strength of the non-social stimuli when presented with social stimuli and alone, (b) assessing preference for non-social stimuli in a choice arrangement with both human and non-human social stimuli, and (c) employing nonspecialized equipment, rather than complex eye-tracking technology, since the tablet computer and application are readily available, portable and easy to use.

As found in previous research^5,6^ we found that children with ASD showed a preference for the non-social geometric images when presented concurrently with human social images. Similar to some previous findings^13^, but in contrast to others^5,6^, the TD children did not show an overall preference for human social images, rather there was almost equal responding to the two types of stimuli. This may be explained by an increasing interest in non-social geometric images seen in ASD and TD children with age^6^. In addition, this may be due to the social stimuli being faces rather than more complex social stimuli, such as children playing, which may be more interesting to TD children as they get older and less interesting to children with ASD^28^. When presented with a choice between the non-social images and films of dogs, we found the children with ASD preferred the non-social images. Interestingly, the results of the current studies differ from previous research examining interest in humans, dogs and objects where children with ASD were found to prefer dogs to objects and humans^20,21^. This could be because certain non-social stimuli, for example moving images, are more reinforcing than other types of non-social stimuli, e.g. household objects and toys, and therefore continue to be reinforcing in the presence of alternative stimuli. The non-social images in the current study maintained their reinforcement valence in Study 3 in the absence of other stimuli which suggests the moving non-social stimuli is particularly powerful.

When non-social stimuli are more reinforcing than social stimuli, non-typical development may occur in at least two important ways: First, infants with a preference for non-social stimuli will attend to such stimuli (e.g., geometrical patterns in the environment, moving objects, flickering lights, nonverbal sounds) at the cost of attending to social stimuli, such as the eyes and voices of caregivers. As the infant grows older and acquires a more advanced motor repertoire, non-social reinforcers will not only select attention, they will also select repetitive and stereotyped behaviors (e.g., hand flapping, object twirling, lining of objects, playing the same YouTube video or computer game over and over again), because these behaviors are motivated and reinforced by the sensory consequences they produce^29,30^. Secondly, in infants who show increased preference for non-social stimuli, pragmatic communication, social interests and social skills may become a deficit because attending to the face and eyes of caregivers, listening to human voices, exhibiting joint attention and social communication require motivation for social stimuli^1,2^. In the case of ASD, these social stimuli may be overshadowed or otherwise outcompeted by the highly reinforcing non-social stimuli. One exception is echolalia, which is more often seen in children with ASD. Echolalia does not require the same type of social interests since copying words and sentences may be reinforcing in itself^30^.

This interpretation can be seen as a development of and perhaps a more precise articulation of the social motivation hypothesis, which focuses on the lack of social motivation and a deficit in processing of social rewards, since the current interpretation focuses on the reinforcement strength of non-social stimuli. The social motivation hypothesis does not explain directly the presence of stereotyped, repetitive and restricted behaviors^4^. An elevated effect of non-social reinforcers, however, may explain both the lack of social communication and social interests as well as presence of stereotyped and repetitive behavior which both define ASD^31^. To evaluate this non-social reinforcement hypothesis, research should assess whether increased interest in non-social stimuli occurs prior to, concurrently with, or after the decreased interest in social stimuli. For example, declining attention to eyes has been found to occur between the age of two and six months in infants who later receive a diagnosis of ASD^9^. To support the non-social reinforcement hypothesis of ASD, increased interest in visual non-social stimuli should occur prior to or concurrently with the declined attention to eyes.

Rather than being attractive or neutral, certain non-social stimuli may have aversive functions^32,33^, such as when a child reacts negatively to a vacuum cleaner. In addition, many children with ASD find some social stimuli or social situations aversive^34,35^. When certain stimuli are aversive (social or non-social) the child will behave in ways that effectively removes these stimuli, and sometimes, engaging in aberrant or aggressive behavior is the most effective way to do so^36^. Non-social stimuli functioning as reinforcers and aversive stimuli in the infants’ natural environment are therefore likely to shape behavior characteristic of children with ASD. Identifying which children are susceptible and how those behaviors develop is an important area for future research.

The affinity for non-social reinforcers as seen in individuals with ASD may be explained by differences in their neurobiological makeup. Research has examined neural responses during reward anticipation and reward processing in individuals with ASD, utilizing functional magnetic resonance imaging (fMRI), suggesting hypoactivation across mesolimbic and frontostriatal networks in individuals with ASD. However, for certain non-social stimuli, hyperresponsivity rather than hypoactivation has been found^4,37^. Future research could aim to assess neural responses while processing preferred non-social stimuli, in children with ASD and TD children, as compared to processing of social stimuli. Identifying the neurobiological mechanism underlying increased reinforcement value of non-social stimuli and decreased reinforcement value of social stimuli may help uncover the biological mechanism underlying ASD. Examining neural responding in children whilst taking the app assessment could lead to further insight into the differences between TD infants and those who later receive a diagnosis of ASD.

The results of the present study may have important implications for treatment. Firstly, to increase children’s interest in social stimuli, treatment should focus on establishing eye looking, face reading and listening to human voices as powerful reinforcers^38^. Secondly, treatment should focus on expanding the children’s interest in the type of non-social stimuli that TD children find interesting, such as toys^39^. Thirdly, treatment should focus on reducing stereotyped and repetitive behaviors as well as improving treatments for these behaviors. Finally, as long as non-social stimuli are more reinforcing than social stimuli, effects of interventions targeting social interest, social skills and social language may not be long lasting. As shown in the present study, when left to their own devices, children with ASD engage in behaviors that produce potent non-social reinforcers at the cost of engaging in behaviors that produce social reinforcers. In real life, stereotyped, repetitive and ritualistic behaviors are the type of behaviors that produce such non-social reinforcers and, hence, these behaviors may occur at the cost of engaging in a newly acquired repertoire of social behaviors such as joint attention, social communication and peer play.

Rather than attempting to change this interest in non-social stimuli, one approach could be to focus on understanding the individual’s interest in non-social stimuli and for non-ASD people to accept this interest. This may be effective for some children with ASD and thus allow them to develop and see the world in a way that is natural for them, with others’ accepting and understanding this different viewpoint. Furthermore, interest in certain types of non-social stimuli may be beneficial and lead to a heightened ability in particular areas, for example interest in numbers may result in exceptional mathematical ability. However, for many individuals with ASD an increased interest in non-social stimuli may not be beneficial. Toddlers with autism who have higher cognitive ability and less severe social communication deficits at diagnosis have been found, in adolescence, to have more adaptive skills, less severe autism symptoms and a decrease in social deficits over time when compared to children with a lower cognitive ability^40,41^. Furthermore, research has shown that toddlers with ASD who showed intense fixation on geometric images had the most severe ASD symptoms, fewer language skills, and lowest overall IQ scores^6^. This suggests children with ASD who have the highest interest in non-social reinforcers are typically children, and later adolescents, with fewer skills. Therefore, these individuals may, in the long-term, be unable to function in society or have any form of independence, if some form of treatment is not employed to reduce this interest and increase interest in social reinforcers. Therefore, consideration should be given to developing procedures that reduce the reinforcing power of some non-social stimuli. Future research should consider interest in which types of non-social stimuli is beneficial and interest in which types is detrimental and identify those children that can benefit from such interests and those that may not.

One limitation of the current study is that many of the same participants took part in the three studies and this may have negative effects on the generalization of the results. In addition, while the TD children did not have any concerns raised about their development by the parents or nursery staff, they were not assessed with cognitive measures or for autism symptoms.

Future research could consider some procedural issues. For example, the social stimuli used were Caucasian adults while the participants were of varied ethnicity. Since ethnicity may have an impact on how long children examine faces^28^, including more variation in ethnicity within social stimuli while including color for the non-social stimuli should be a consideration for future research. Also, when choosing stimuli to assess interest in non-social and social images, the participants’ age and ability level should be taken into consideration^4^. For example, interest in complex social stimuli (typically involving several people) seems to increase with age in TD children, while not in children with ASD^42,43^. Also, monetary stimuli should be avoided as individuals with ASD typically show little interest in such stimuli as well as abnormal neural responses to them^4,44^.

## Method Study 1

### Participants

All research protocols for Study 1 and the subsequent two studies were approved by the Norwegian Centre for Research Data (NSD). All research was performed in accordance with relevant guidelines and regulations, and informed consent was obtained from all participants’ legal guardians.

Participants were 27 children (6 girls) with autism spectrum disorder (ASD) and 40 (18 girls) typically developing children (TD). The developmental age of participants with ASD was matched with the chronological age of the TD children. Table 1 shows participant’s gender and chronological age for both the ASD and TD children, and developmental age, cognitive score, adaptive behavior, and autism symptoms for the participants with ASD.

The participants with ASD had (a) a diagnosis of autism based on the ICD-10 criteria^45^ set by a medical professional who was independent of the study, (b) developmental age of five years or below, and (c) no medical conditions that could interfere with the study, such as sustained uncontrollable epilepsy, or major motor or sensory impairments. The participants with ASD were recruited from treatment centers providing comprehensive educational interventions based on ABA (Applied Behavior Analysis). Initial participant contact was made either by email from the first author or in person by a senior staff member unrelated to the study. Full written information regarding the studies was provided in both cases, and parents were informed that participation was voluntary, and not taking part would not affect services to their child or their relationship with the service provider. The first author was available to answer questions via email or telephone, and parents were able to accept by completing a consent form and returning via email, or to decline by email.

The TD participants had (a) no psychiatric diagnosis and no concerns about the child’s development raised by parents or professionals, (b) chronological age of five years or below, and (c) no medical conditions that could interfere with the study, such as sustained uncontrollable epilepsy, and major motor or sensory impairments. The TD participants were recruited either through a nursery (the majority) or via acquaintances of the first author. Written information was provided to all the parents and those that wished to participate sent a completed consent form to the nursery or to the first author, who was available to answer questions via email or telephone. Parents were informed that participation was voluntary and declining to participate would not affect their relationship with the nursery or the first author.

### Tablet assessment of non-social and social interest

Participants’ interest in non-social and social stimuli was assessed using applications (apps) designed for the purpose of this study, presented on a Samsung Galaxy Tablet (See Fig. 1). The non-social stimuli consisted of six different films of abstract moving geometric patterns in black and white, the type of stimuli often used as screen savers. The stimuli were created by David Szakaly (artist name: davidope) and downloaded from the internet. Social stimuli were six different films of the face of a young adult smiling and talking or doing peek-a-boo. During each session, one of the six non-social films and one of the six social films (size 7 cm × 7 cm per image) were presented side by side on the screen. The pairs were presented in either one of two random slots of three positions across the screen; left, center, and right. The location of the non-social and social stimuli was randomized within the pair, so that each stimulus appeared on either the left or the right of the pair randomly. Both films were pixelated and appeared blurred. After five seconds if a blurred film was not touched, then the screen changed to present another pair of blurred non-social and social films, and five seconds later a third pair were presented, and so on. One session was 90 seconds duration and 8 sessions were conducted with each child. No auditory stimuli were presented at any time. During the sessions, whenever a blurred film was touched then it was interrupted, and that film became clearly visible increasing to a size of 13 × 13 cm and displayed in the center of the screen for two seconds. Once the video-image was viewed for two seconds, the screen returned to a new pair of blurred films and the session continued until the 90 seconds’ duration was completed. The child could touch the blurred films to view the clear films as frequently as they chose during the 90 second sessions. The app recorded the frequency of touches on each image for each of the eight sessions. The eight sessions were administered within three hours for all children, and the child was given breaks between sessions appropriate for their level of attention and ability.

The app assessments were conducted in the child’s home, nursery or clinic setting. The child was seated where comfortable (e.g., on a chair, sofa or the parent’s knee). The tablet was placed on a table or on the child’s knee in such a way that the child was able to attend to it and touch it. The child was given an instruction to touch what they wanted on the screen of the tablet.

The app was administered by the first author, senior staff from the service provider, or a Board Certified Behavior Analyst (BCBA). For a minority of the participants, reinforcement (praise or food) was provided intermittently contingent on sitting and paying attention to the screen. No reinforcement was provided when the child touched the screen or when the selected video was playing. Training for administering the app was provided by the first author. Training was given through step by step demonstration of the app with verbal instruction and accompanying written procedures to read and follow during training. Trainees then demonstrated the procedures within the training setting and were provided with the written procedures to use during administration with the child.

### Assessment of intellectual functioning

Participants with ASD were assessed using either the Bayley Scales of Infant and Toddler Development (Third Edition)^46^, the Wechsler Preschool and Primary Scale of Intelligence (WPPSI-IV)^47^, the British Ability Scales (BAS-3)^48^ or Psycho-Educational Profile–Revised (PEP-R)^49^.

The WPPSI-IV was administered with those participants within the age range for the assessment (2.6–7:7 years), and who were able to achieve a basal on four out of five subtests (for age range 2:6–3:11) or five out of six subtests (for age range 4:0–7:7). Only those subtests required to calculate a full-scale intelligence quotient composite score (FSIQ) were administered.

The cognitive subtest of the Bayley Scales was administered to participants below 2:6 or who were unable to achieve basal on the WPPSI-IV. If the participant was above 42 months chronologically and the Bayley Scales had been administered, then a cognitive ratio IQ score was calculated (chronological age divided by developmental age multiplied by 100), using the child’s developmental age, as identified on the Bayley Scales.

Most participants were assessed by the first author or a consultant from the service provider using the WPPSI-IV (3 children) or the Bayley Scales (18 children). Two of the participants had been assessed within the last six months by an independent educational psychologist using the BAS-3, therefore this score was used for those participants. Three children were receiving services from another service provider and therefore were assessed using the PEP-R. Cognitive score for one child was not available.

### Assessment of adaptive behaviors

Children’s adaptive skills were assessed with the Vineland Adaptive Behavior Scales, Second Edition (Vineland-II, Survey Form)^50^. The Vineland yields standard scores for communication, daily living skills, socialization and motor skills, as well as a composite standard score. The Vineland-II was administered by either the first author, consultants from the service provider or a Board Certified Behavior Analyst (BCBA).

### Assessment of autism symptoms

The Childhood Autism Rating Scale (CARS-2)^51^ was completed for participants with ASD. The CARS2 is a 15-item rating scale used to identify children with autism. The CARS-2 was administered by either the first author, consultants from the service provider or a Board Certified Behavior Analyst (BCBA).

### Data analysis

Number of presses to access non-social and social stimuli were counted for each participant, and further analyses used the proportion of presses on non-social stimuli. Proportion of social responding can be identified as 100% minus proportion non-social responding. Group difference between the ASD and TD groups were investigated using an independent samples Welsch *t*-test. Also, an effect size for the group difference was computed, using Cohen’s *d*. Pearson correlations were used to investigate any covariation between percentage non-social presses and developmental age, autism symptoms, cognitive ability, adaptive behavior and subscales of the Vineland Adaptive Behavior Scales. All statistical analyses were conducted using JASP^52^.

## Method Study 2

### Participants

The ASD group included 19 participants (4 girls), and the TD group 21 participants (10 girls). Mean developmental age in the ASD group was 29.9 months (range 12–50 months) and mean chronological age in the TD group was 33.6 months (range 17–63 months) (See Table 1).

All participants from Study 1 were invited to participate in Study 2; in the ASD group, 18 of the 19 participants agreed, in the TD group, 17 of 21 agreed.

### Measures, procedures and analysis

All measures and procedures were identical to Study 1, except that the films of human faces were replaced with films of dogs’ faces (see Fig. 1d for an example of the dogs’ stimuli). The dogs were filmed in front of a plain white wall, with the dog’s face visible to the camera. The non-social stimuli and the dogs’ faces were blurred when presented on the tablet app, in the same way as the films in Study 1. Assessments of intellectual functioning were conducted identically to Study 1 except one child, who was not in Study 1, was assessed using the Batelle Developmental Inventory (BDI-2)^53^ as he received services from a different service provider. Statistical analyses were carried out identically to that of Study 1.

## Method Study 3

### Participants

The ASD group included 17 participants (5 girls), and the TD group 23 (13 girls) participants. Mean developmental age in the ASD group was 32.2 months (range 12–50 months) and mean chronological age in the TD group was 34.9 months (range 17–60 months) (See Table 1).

All participants from Study 1 and 2 were invited to participate in Study 3. In the ASD group, all 17 participants from Study 3 participated in Study 1, and 14 children participated in Study 2. In the TD group, 22 of 23 children participated in Study 1, and 10 participated in Study 2.

### Measures and procedures

Measures were those described in Study 1 except that a different application on the Samsung tablet was used. The stimuli use in the application were identical in type and size to those used in Study 1, however the app was designed so that only one type of the blurred stimuli (non-social or social) was presented on the screen during a session. If untouched, the blurred film moved between the left, center or right position randomly every five seconds. To view the film clearly the child was initially required to touch the blurred film twice. Once the clear film had been viewed twice at that ratio, the schedule increased by two. For example, initially the child touched the blurred film twice consecutively, and viewed the clear film. Once viewed, it returned to a blurred film in a different position on the screen. Two more consecutive touches on the blurred film were required to view the clear film a second time. At this stage, the schedule increased to four consecutive touches before the clear film could be viewed. The schedule remained at this level until the clear film had been viewed twice. Following this the schedule again increased by two, therefore the child was then required to touch the blurred film six times to view the clear film, and so on.

Touches outside of the blurred film led to no visible consequence, however the ratio counter would begin again. For example, if the schedule was two and the child touched the blurred film once, then touched outside the screen, they would have to touch the blurred film two more times before they could view the clear film. As such, touches within the blurred film were required to be consecutive to view the clear film. Touches outside the blurred film in between viewings did not affect the change in ratio schedule. Following a clear viewing, the screen returned to the blurred film and the session continued.

One session of the non-social stimuli and one session of the social stimuli were presented to each child. The order of presentation of non-social and social stimuli was counterbalanced across participants. Sessions ended when the child failed to respond for 60 seconds therefore the length of session and number of touches was dictated by the child. Once the first session presenting one stimulus type (e.g., non-social) ended, the child was given a break for between 30 and 60 minutes before the second session presenting the second stimulus type (e.g. social) was administered.

Assessments of intellectual functioning were conducted identically to Study 1

### Data analysis

Reinforcement value was measured in three ways: (a) by frequency of responses, (b) session length before extinction (seconds), and finally, (c) break point, (reinforcement schedule in effect at the time of extinction). For these three metrics of reinforcement value, the proportion of non-social responses out of the total for both sessions (sum of touches, sum of session lengths, and sum of break points) was computed. Independent samples t-tests were used to compare group differences between ASD and TD. Pearson correlations were used to investigate whether any background variables correlated with proportion of non-social break-points.

## Supplementary information

Pearson r

## Data Availability

The datasets generated during and/or analysed during the current study are available from the corresponding author on request.
