# Supplementary material for: Children with Autism show Atypical Preference for Non-social Stimuli
Source: Sci Rep. 2019 Jul 17;9:10355. doi: 10.1038/s41598-019-46705-8 (PMC6637109; doi:10.1038/s41598-019-46705-8)
Supplement: Supplementary file 1 — Pearson r [file 41598_2019_46705_MOESM1_ESM.docx]

Children with Autism show Atypical Preference for Non-social Stimuli

Catherine M. Gale^1^, Svein Eikeseth^1^* and Lars Klintwall^2^

^1^Oslo Metropolitan University, ^2^Stockholm University

Supplementary Materials

Table 1

*Unprotected Pearson Correlations of Assessment Scores with*

*Nonsocial Stimuli Responding for Participants with ASD*

­­­­­­­­­______________________________________________________

Study 1

Developmental Age (months) 0.233

CARS -0.115

Cognitive Score -0.187

Vineland Adaptive Behaviour Scales:

Adaptive Behaviour Composite 0.147

Communication 0.128

Daily Living Skills 0.160

Socialization 0.035

Motor 0.000

Study 2

Developmental Age (months) 0.151

CARS -0.170

Cognitive Score -0.302

Vineland Adaptive Behaviour Scales:

Adaptive Behaviour Composite 0.172

Communication 0.200

Daily Living Skills 0.025

Socialization 0.055

Motor 0.277

Study 3 - Touches

Developmental Age (months) -0.256

CARS 0.269

Cognitive Score -0.372

Vineland Adaptive Behaviour Scales:

Adaptive Behaviour Composite -0.270

Communication -0.428

Daily Living Skills -0.082

Socialization -0.301

Motor -0.268

Study 3 - Duration

Developmental Age (months) -0.214

CARS 0.258

Cognitive Score -0.263

Vineland Adaptive Behaviour Scales:

Adaptive Behaviour Composite -0.324

Communication -0.430

Daily Living Skills -0.123

Socialization -0.304

Motor -0.309

Study 3 – Break Point

Developmental Age (months) -0.120

CARS 0.148

Cognitive Score -0.253

Vineland Adaptive Behaviour Scales:

Adaptive Behaviour Composite -0.062

Communication -0.184

Daily Living Skills 0.029

Socialization -0.287

Motor 0.055

______________________________________________________

*Note*. All correlations were statistically nonsignificant.
